# Alcohol‐related brain damage: A mixed‐method evaluation of an online awareness‐raising programme for frontline care and support practitioners

**DOI:** 10.1111/dar.13545

**Published:** 2022-09-12

**Authors:** Rebecca Ward, Gareth Roderique‐Davies, Harriet Hughes, Robert Heirene, Simon Newstead, Bev John

**Affiliations:** ^1^ Faculty of Life Sciences and Education University of South Wales Pontypridd UK; ^2^ Department of Psychology Swansea University Swansea UK; ^3^ College of Health and Human Sciences Charles Darwin University Darwin Australia

**Keywords:** alcohol‐related brain damage, alcohol‐related dementia, alcohol‐related neurocognitive disorders, awareness‐raising, public health intervention

## Abstract

**Introduction:**

Alcohol‐related brain damage (ARBD) is an umbrella term referring to the neurocognitive impairments caused by excessive and prolonged alcohol use and the associated nutritional deficiencies. This study evaluated the outcomes of an online research‐informed training program for ARBD which aimed to improve client outcomes by promoting support staff's awareness and confidence in working with clients who may have (or who are at risk of developing) the condition.

**Methods:**

Staff working within a large non‐governmental non‐profit housing organisation (*n* = 883) enrolled in the training program. Questionnaires were used pre‐ and post‐training to collect self‐reported awareness of ARBD and confidence in supporting individuals with the condition. Semi‐structured interviews were conducted with 27 staff members approximately 10 weeks post‐completion of the program. Interviews were audio‐recorded, transcribed verbatim and analysed by employing qualitative content analysis.

**Results:**

Findings from the questionnaires indicated a significant increase in all measures after completing the training program. Three main themes were developed based on the interview data: changes to awareness and understanding; professional practice; and training‐specific characteristics. Participants reported changes in their ability to identify potential service users with ARBD and confidence in doing so.

**Discussion and Conclusion:**

Our findings demonstrate that online training programs can be effective in improving support staff's ability to identify ARBD, potentially leading an increase in signposting service users to relevant services. The research‐informed nature of the training demonstrates that translating research findings directly to frontline workers can have a substantial impact and may improve outcomes for this client group.

## 1 INTRODUCTION

Prolonged and excessive alcohol consumption has a significant impact on health and wellbeing, with evidence suggesting that around 1 in 20 deaths are attributable to the harmful use of alcohol [1, 2, 3]. At the same time, the long‐term impact that alcohol has on the brain specifically is not as widely known [4]. Alcohol‐related brain damage (ARBD) is an umbrella term used to describe a set of related conditions caused by chronic alcohol use combined with associated nutritional deficiencies. ARBD prevalence estimates range between 0.034% and 1% of the population [5, 6, 7], with areas of high socio‐economic deprivation and specific at‐risk populations such as the homeless reporting higher rates [5, 7]. For example, a study of homeless hostel dwellers in Glasgow reported a rate of 21%, while around 82% of the hostel dwellers in the sample displayed evidence of some cognitive impairment [5]. Females may also be disproportionately affected by ARBD as they appear to develop ARBD at a significantly younger age than males after consuming harmful quantities of alcohol for a shorter period of time [6, 8]. In Wales specifically, recent research has calculated the age‐specific prevalence of ARBD as 34 cases per 100,000 people [6], although the authors note these figures are likely to be an under‐estimation of the true number of individuals with the condition.

ARBD is a substantial public health concern, and yet research suggests that there is currently a lack of knowledge and understanding of ARBD amongst both the general public and, perhaps more importantly, health‐care professionals [9, 10]. This is reported to have resulted in individuals going undiagnosed and, in some cases, misdiagnosed [11, 12, 13] and therefore untreated [14, 15]. There is evidence that ARBD is not a degenerative condition and research also suggests that not only can it be prevented with adequate support, but also that those with the condition have the potential to improve and even reverse their symptoms if recognised early in the disease trajectory and managed promptly [16]. If left untreated, reports suggest that around 80% of those with the condition will develop severe and permanent neurological disorders [17], although limited information exists on this issue as the identification of untreated cases of ARBD is difficult.

Stigma is often synonymous with alcohol use disorder and those with ARBD face further stigmatisation due to their cognitive impairments, often being viewed as non‐compliant or problematic [10]. A lack of engagement with services and failure to attend appointments may in itself be an indicator of the condition, highlighting the crucial relationship between a lack of awareness and understanding of ARBD by service providers, and barriers to appropriate treatment [9].

Although hazardous and harmful alcohol consumption is considered a worldwide concern [3], within the UK context, Wales is currently at the forefront of legislating health‐related protections relating to harmful drinking. In 2014, Public Health Wales recognised ARBD as a significant public health concern [18] and the Welsh Government has since prioritised the issue of harmful drinking. South Wales, in particular, is one of the most socio‐economically deprived post‐industrial areas of the United Kingdom, with one of the highest rates of deaths attributable to alcohol [19]. In partnership with Public Health Wales, some members of the present research team (Bev John, Gareth Roderique‐Davies and Robert Heirene) co‐developed the Welsh Government's Substance Misuse Treatment Framework for ARBD [20]. One primary recommendation from this strategic document is to improve awareness and understanding of ARBD.

As a direct response to this recommendation, along with the high prevalence of ARBD in Wales [6] and an absence of recognised training currently available, an evidence‐based training program was developed and delivered by the present research team (see Section 2). This was targeted at health and social care support staff who were considered to be the most relevant sector who are likely to have contact with individuals who have undiagnosed or misdiagnosed ARBD [9]. The training was designed to increase staff's confidence, awareness and knowledge of ARBD.

### 1.1 Training program development

The initial stage in developing the training program was to review a range of up‐to‐date research relating to ARBD [10, 21], including research conducted by members of the present research group [6, 9, 22]. Consultations with a range of health and social care professionals were then undertaken (by RW) to ascertain the training needs of staff working in this sector. These included one‐to‐one interviews and group meetings with service managers and higher managerial staff to discuss any current training provisions and the range of services that might benefit from the training. Consultations with learning and development specialists and software experts were also undertaken to provide guidance on developing online training resources. Following this, the materials were developed by members of the present research team (RW, G‐RD, BJ and SN), which were underpinned by recent research findings [10, 21] and guided by information obtained from the consultations. Next, all materials were reviewed by several external partners, including Consultant Psychiatrists and Alcohol Change (an Alcohol Information Support Charity) and adapted accordingly. Prior to launching the training, our partner organisation piloted the training to ensure that all elements of the program were working correctly. Three members of staff from different branches of the organisation completed the training and reported back any issues and suggestions.

### 1.2 Training program description

The multi‐level training program was delivered online and included two self‐guided e‐Learning modules, information leaflets, handbooks and videos from ARBD specialists. Each e‐Learning module had three sections with the content focussing on: (i) identifying ARBD; (ii) the prevalence and characteristics of ARBD; and (iii) the importance of treatment and early intervention. After each section of the e‐Learning modules there was a recap of the key take‐home messages. The e‐Learning modules had an optional voiceover and consisted of interactive elements, scene‐based slides and information sections (see Data S1, S2 and S3, Supporting Information). Information was provided about how to recognise the signs and symptoms of ARBD; how to support someone with, or at risk of developing the condition; and how the condition is treated by specialists. Successful completion of the training required individuals to complete a 10‐question quiz based on the content of the program. A pass mark of 80% or above was required, although the training allowed as many attempts as was necessary. The training could be reviewed at any stage. Electronic leaflets, handbooks and videos were available to access through the training program itself and on the organisation's internal learning portal. The training program has since been endorsed by the Royal College of Psychiatrists in Wales. For further information, including example screenshots of the training, see Data S1, S2 and S3.

The aim of the current study was to evaluate immediate changes and the impact of the training program on several outcome measures within health and social care support staff in Wales. The research questions were:

1. How effective is the ARBD training program in increasing immediate awareness and understanding of ARBD?
2. How successful is the ARBD training program in increasing the participants self‐reported ability to identify the signs and symptoms of ARBD, and in improving their understanding of treatment options for ARBD?
3. How effective is the ARBD training program in enhancing participants' confidence in supporting someone with ARBD?
4. What perceptions do participants have of the training program?

## 2 METHODS

The training program was launched in October 2020 and all staff employed by the partner housing organisation (see Section 2.1 below for more details) were able to access the training. Enrolment in the training program is ongoing, although here we report outcomes from all staff who completed the training up to November 2021. A mixed‐methods data collection approach was used to answer the above research questions. This included a combination of self‐reported pre‐ and post‐training measures of awareness, understanding and attitudes towards ARBD (see Section 2.2 below for more information), and interviews capturing detailed information about participants' experiences of undertaking the training. A mixed‐method design provided an insight into the impact of the training on our key outcomes (i.e., awareness and understanding of ARBD) while allowing for an in‐depth exploration of individual and collective experiences. As the training program and evaluation was delivered during the COVID‐19 pandemic, all training and follow‐up interviews were conducted online.

### 2.1 Participants

The organisation who collaborated on the development and dissemination of the training program was a large not‐for‐profit housing organisation in Wales. This organisation employs over 2000 staff members and supports around 10,000 individuals who may be at risk of ARBD. The organisation offers support for a wide range of needs, including homelessness support, substance misuse services and residential care for older clients. Eight hundred and eighty‐three individuals working within this organisation's support services enrolled on the ARBD training program. Of these, 812 (91.96%) completed the full program (see Table 1). All of these responded to all questions in the evaluation questionnaire.

**TABLE 1:** Demographic information of the participants included in the quantitative and qualitative analysis

Demographic information	Pre‐ and post‐data	Evaluation interviews
	*N* (%)	*N* (%)
Role		
Frontline staff	618 (76.11)	13 (48.15)
Scheme support officer	40 (4.93)	4 (14.81)
Manager	60 (7.39)	4 (14.81)
Area manager	10 (1.23)	0 (0.00)
Support worker	26 (3.20)	5 (18.52)
Other	58 (7.14)	1 (3.70)
Prior awareness of ARBD		
Yes	480 (59.11)	20 (74.07)
No	332 (40.89)	7 (25.93)
Gender		
Male	222 (27.34)	8 (29.63)
Female	586 (72.17)	19 (70.37)
Prefer not to say	4 (0.49)	0 (0.00)

Abbreviation: ARBD, alcohol‐related brain damage.

Following the training, managers within the organisation were contacted to inform them of the opportunity to participate in follow‐up evaluation interviews via video conferencing software. Interviews were conducted approximately 10 weeks after staff had engaged with the training program. Follow‐up interviews were conducted with 27 participants over 18 interview sessions. Interviews provided an opportunity for trainees to share more detailed accounts of their experience with the training.

### 2.2 Measures

In order to evaluate the immediate change in awareness, understanding and attitudes, an evaluation was in‐built to the training program to capture information relating to trainees' pre‐ and post‐training awareness, understanding and attitudes towards ARBD (see Table 2 for the full wording of questions). These self‐reported questions were a requirement of completing the training program. Prior to completing the training, trainees were asked if they had any prior awareness of ARBD and to rate their current level of awareness, understanding and ability to identify ARBD on a five‐point Likert scale. Trainees were also asked to rate their ability to identify signs and symptoms, their understanding of treatment possibilities and their confidence in supporting someone with ARBD, again on a five‐point Likert scale. Upon completion of the training, a second questionnaire captured information relating to the self‐reported ratings for the same measures as described above. In addition, nine further questions were asked, to ascertain participant attitudes towards ARBD, their satisfaction with the training, the effectiveness of the program, as well as an opportunity to provide further feedback.

**TABLE 2:** Pre‐ and post‐outcome measures

Item	Pre‐training	Post‐training	*t*	*p*	Mean difference	95% CI
Awareness of ARBD	**2.49 (1.01)**	**4.09 (0.75)**	**39.67**	**<0.001**	**1.61**	**1.54–1.70**
Understanding of ARBD	**2.43 (0.97)**	**4.09 (0.76)**	**51.88**	**<0.001**	**1.68**	**1.60–1.76**
Ability to identify someone at risk of ARBD	**2.42 (1.02)**	**3.99 (0.77)**	**41.66**	**<0.001**	**1.60**	**1.53–1.68**
Ability to identify signs and symptoms of ARBD	**2.28 (0.96)**	**4.03 (0.77)**	**47.11**	**<0.001**	**1.77**	**1.70–1.85**
Understanding of treatment possibilities for ARBD	**2.12 (0.91)**	**4.08 (0.76)**	**42.66**	**<0.001**	**1.96**	**1.89–2.03**
Confidence in supporting someone with ARBD	**2.44 (1.06)**	**4.06 (0.79)**	**39.94**	**<0.001**	**1.63**	**1.55–1.71**

*Note*: Self‐reported ratings reported as means with standard deviation in parenthesis. Bold indicates a significant effect with *p* < 0.001. Likert scale scores ranged from Poor (1) to Good (5) with participants asked, ‘please rate the following’. Calculations adjusted for multiple comparisons using Bonferroni corrections.

Abbreviations: ARBD, alcohol‐related brain damage; CI, confidence intervals.

Follow‐up interviews employed a semi‐structured approach using an interview guide (for the full interview guide, see Supporting Information). This guide was designed to provide structure to the interviews and was developed by consulting recent literature, reflecting on the research aims and considering the training program itself. This led to a focussed but flexible interview approach, while enabling the interviewer to guide the direction of the interviews according to the direction of the discussions. For example, participants were asked about whether the content of the training stimulated any changes to their awareness of ARBD that was relevant to their role.

A combination of one‐to‐one interviews and focus groups were employed as this approach meant that trainees working within the same service could provide a collective account of undertaking the training. This also offered flexibility for some individuals to share their views on a one‐on‐one basis. Integrating both interview and focus group data provided a rich account of individual experiences while also reflecting the nature of the services that participants worked within. Interviews were conducted online via Microsoft Teams and were recorded to support data analysis. Most interviews were conducted on a one‐to‐one basis (*n* = 12), to enable greater flexibility with regard to the direction of the discussion. Four focus groups included two participants, one interview had three participants and one interview included four participants.

The duration of the interviews ranged from 11 to 47 min (*M* = 24.30, SD = 10.44). Participation was entirely voluntary and participants were provided with a study information sheet and were required to give informed consent prior to the interview. Full ethical approval was obtained prior to the evaluation interviews from the Faculty Ethics Committee at The University of South Wales (approval number 20RW11LR).

### 2.3 Data analysis

In order to analyse the quantitative data collected from the pre‐ and post‐self‐reported ratings, a repeated measures multiple analysis of variance was employed. A series of follow‐up paired student *t*‐tests were conducted on all outcomes where necessary, using Bonferroni corrections to correct for multiple comparisons. Assumptions of normality were met by using Q–Q plots and checking skewness and kurtosis. Statistical analyses were undertaken using SPSS and Jasp.

To analyse the qualitative data collected from the interviews, qualitative content analysis was utilised as this approach provides an objective and systematic avenue to describe and quantify the concept being investigated [23, 24]. This method enables a systematic, transparent and reproducible approach to data analysis which includes the process of creating a conceptual model, comprising of categories and concepts that relate to the research phenomenon under study and is often used in health‐related research [10, 25].

In the first steps of the qualitative analysis familiarisation with the data was conducted and a subset of the data was independently coded by the first author (RW) by identifying and defining units of analysis. A segment of text was considered a unit of analysis if it was substantial enough to be considered a whole concept while being definitive in that the same segment did not overlap with a similar concept within each main theme. Similar codes were then grouped into subcategories of common themes. These were refined and structured within the main themes, whereby the main themes were the overarching categories of meaning. Any redundant or duplicate subcategories were merged or removed where appropriate. This led to the initial coding framework which was then used by the second independent coder (HH) to double code the same sections of data.

**FIGURE 1:**
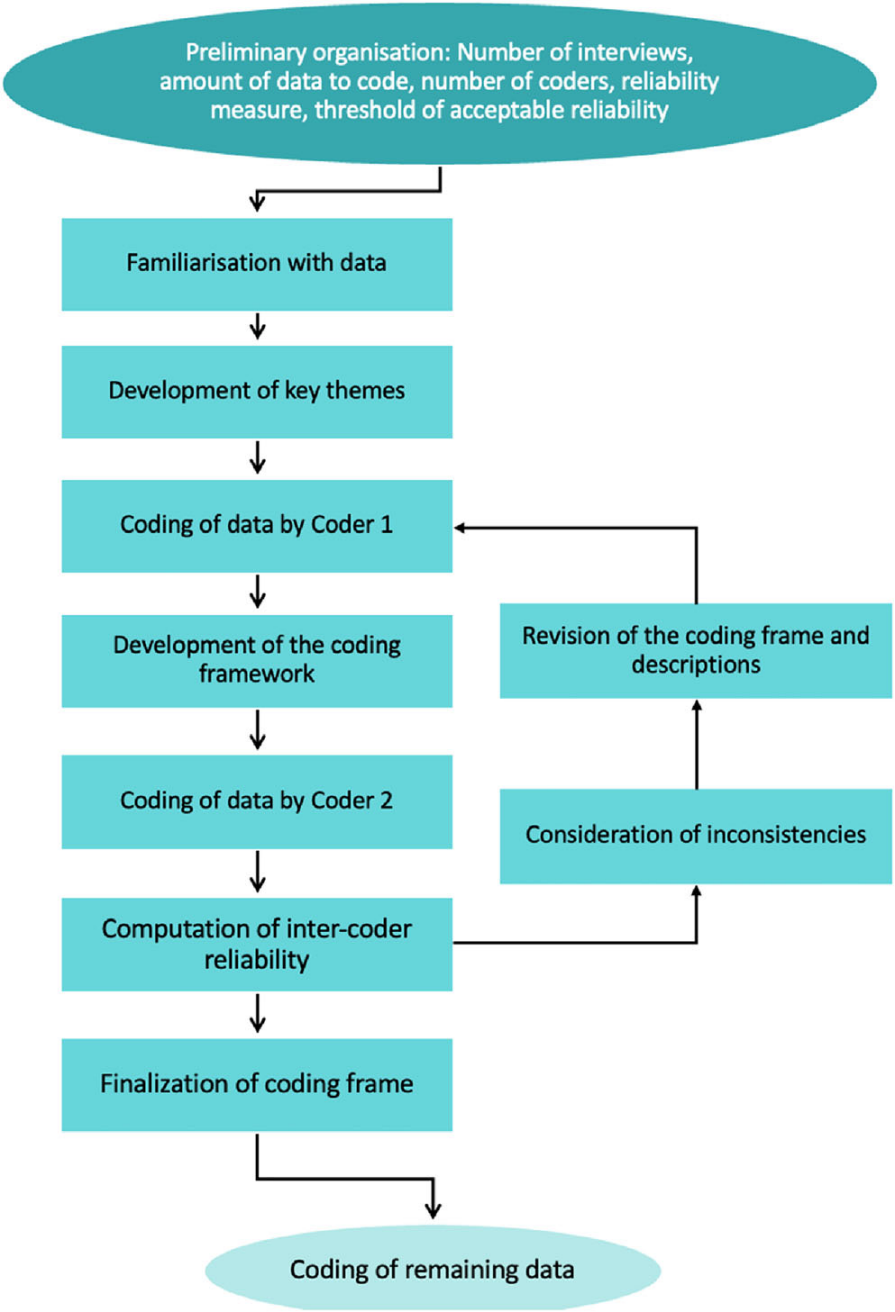
Overview of the coding process

Calculations of intercoder reliability were determined by adopting Cohen's Kappa coefficient. Any inconsistencies between coders were discussed, a common understanding of each code was formulated and the coding frame was refined. The revised coding frame was applied and this process was repeated until an acceptable intercoder reliability rate was achieved (i.e., >70%; [26]). The remaining interviews were coded with 44% of the interviews being independently double‐coded (with at least 25% typically required to satisfy intercoder reliability; [27]). See Figure 1 for a summary of the coding process. Initial reliability after the first round of coding was *k* = 0.58 which increased after the second round of coding to *k* = 0.72. High intercoder reliability was demonstrated utilising the final coding framework with *k* = 0.78.

**FIGURE 2:**
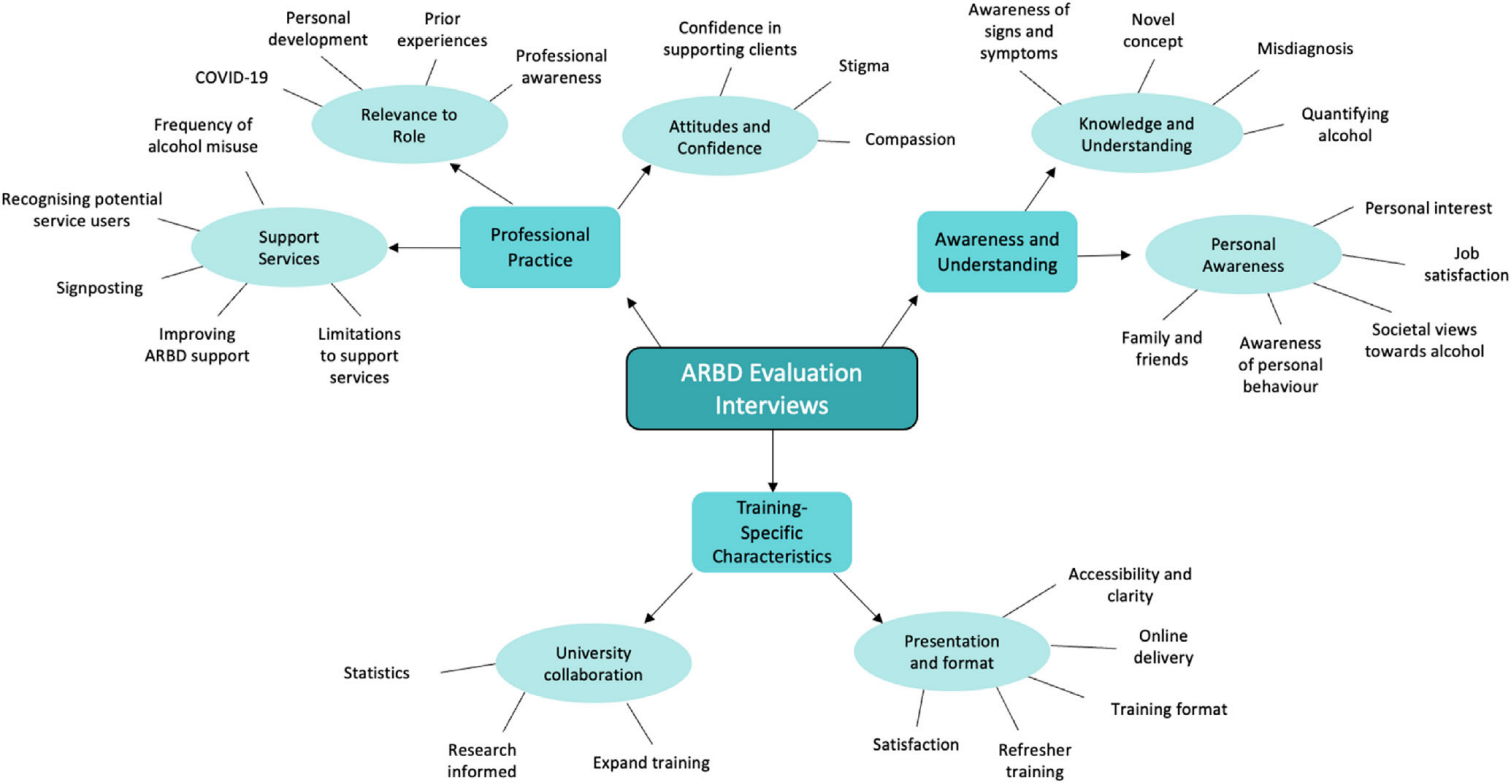
Main themes and sub‐themes that represent the data collected during the evaluation interviews

The final coding frame consisted of three main themes: (i) changes to awareness and understanding; (ii) professional practice; and (iii) training specific characteristic. These main themes had two or three sub‐themes (see Figure 2 for a summary of the overarching main themes and sub‐themes).

## 3 RESULTS

An integrated summary of both the quantitative and qualitative results are presented below, which are organised by each of our research questions.

### 3.1 How effective is the ARBD training program in increasing immediate awareness and understanding of ARBD?

**FIGURE 3:**
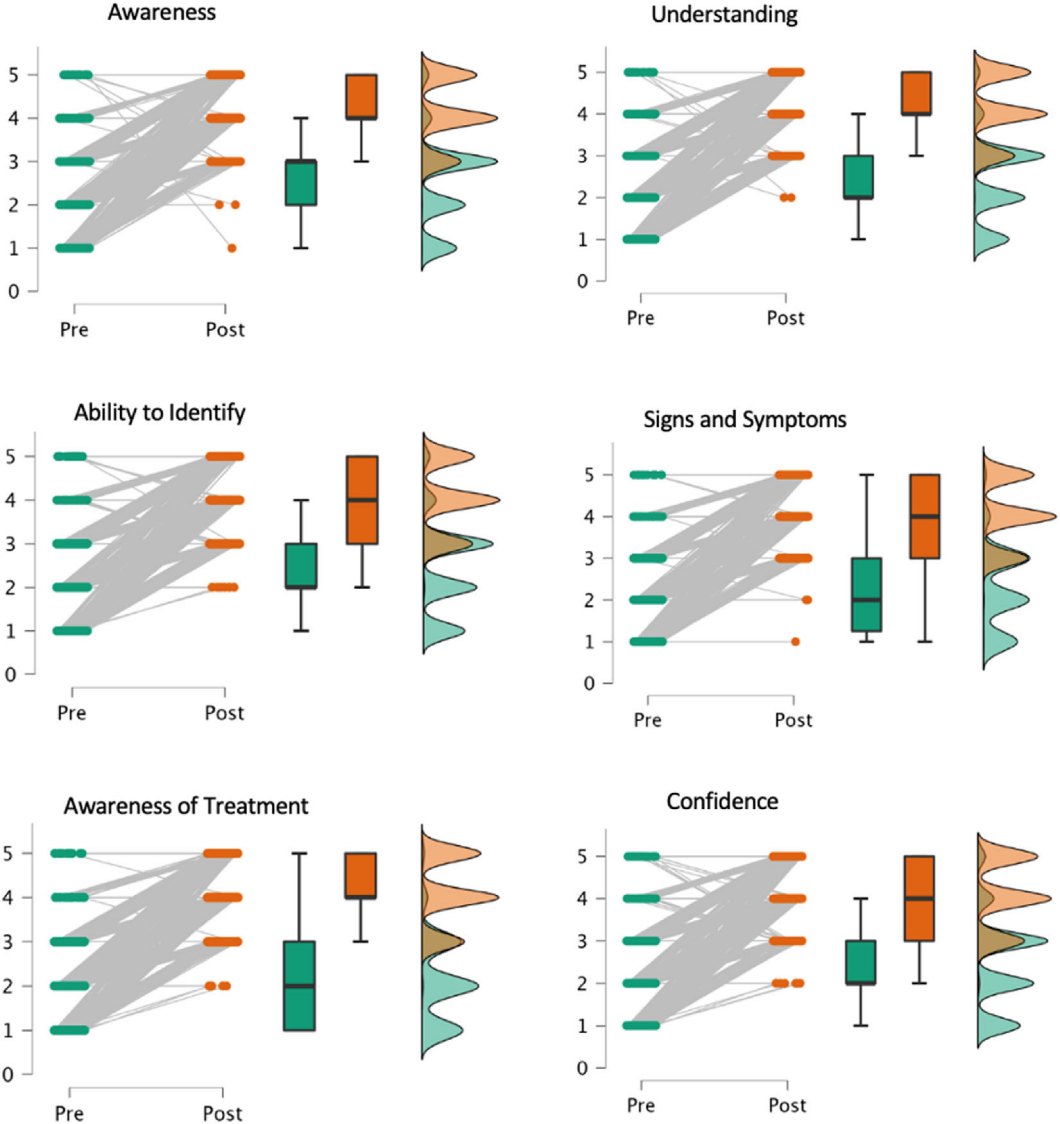
Pre and post self‐reported ratings for all measures presented as data points, boxplots and density plots. Trainees were asked to report their pre‐ and post‐training ratings on a five‐point Likert scale. Participants were asked to rate: ‘Your awareness of ARBD’, ‘Your understanding of ARBD’, ‘Your ability to identify someone at risk of ARBD’, ‘Your ability to identify the signs and symptoms of ARBD’, ‘Your understanding of the treatment possibilities for ARBD’ and ‘Your confidence in supporting someone with ARBD’.

A repeated measure multiple analysis of variance found that the ARBD training program had a statistically significant effect on self‐reported measures across time (pre‐ vs. post‐training), *F*(1, 809) = 2407.764, *p* < 0.001. Follow‐up *t* tests were conducted for each self‐reported measure, which found a significant increase in trainee's awareness *t*(809) = 36.67, *p* < 0.001 and understanding of ARBD *t*(809) = 43.18, *p* < 0.001 (see Figure 3 for a summary of pre‐ and post‐self‐reported outcomes). These are reported with unstandardised effect sizes in the form of mean differences with 95% confidence intervals in Table 2.

Complementing this were reports made by participants during the interviews that they had experienced changes to their awareness and understanding of ARBD. Participants reported that they may now be able to recognise possible signs of undiagnosed ARBD. For example, it participants stated that signs such as missing appointments or the inability to plan would be considered, whereas these might have previously been missed. Many also noted that ARBD was a novel concept to them and, although participants expressed that they frequently worked with people drinking at harmful rates, many commented that the training had brought a new awareness to the condition that they did not have before. Given that many of the participants had no prior knowledge of ARBD, concerns were raised that service users might have been misdiagnosed in the past or that service users may go a long time before receiving a referral and diagnosis.

*‘This course, it makes you wonder if they have been misdiagnosed … it just makes you wonder how long has the misdiagnosis been going on for’*.

A number of participants mentioned the potential overlap of ARBD with other conditions, particularly forms of dementia such as Alzheimer's or early‐onset dementia. These participants expressed their concerns that this may mean that these service users are not currently getting the correct support and treatment for their condition, which may be exacerbated by people feeling ashamed of disclosing information about their drinking habits and the associated stigma.

*‘I kind of just wondered when I was watching how many people are misdiagnosed with dementia and maybe because they have played down how much alcohol they drink and 'cause people don't they don't divulge all their lifestyle choices and I just wonder how many people are not being recognised with that because you know there then you know, people think they've got dementia’*.

Relatedly, some participants noted that ARBD can be misdiagnosed due to the high comorbidity with other issues, such as mental health issues, meaning that this would make it even harder for them in their role to be able to identify service users who may be showing signs of ARBD. Participants also commented that the training had assisted them with understanding standard drink units, what the recommended ‘safe’ limits are, and what is considered ‘harmful’ and ‘hazardous’ drinking. It was noted that this is something that frontline staff and the general public may not be aware of, but that would potentially be valuable for them to know.

‘*That's just stuck in my brain (hazardous drinking units), because all of mine* [clients] *have done that and are doing that, you know that are alcohol drinkers 'cause it's very easily done … Yeah, what scared me is you can also have people who don't really perceive themselves as having an alcohol problem*’.

In addition to improving participants' knowledge and understanding of ARBD on a professional level, several participants noted that the training program also influenced their personal awareness. Firstly, participants highlighted that the training provided a new insight into their own personal behaviour surrounding alcohol use. Many expressed that they had become more conscious of their own drinking habits, the patterns relating to drinking at home and how alcohol may be used as a ‘coping mechanism’.

‘*I found it very useful you know, both on a professional level, obviously to be able to recognise the symptoms may be in clients but also on a personal level like … I'm conscious of the fact that you know a question I often ask myself is do I drink too much or do I drink too often?*’

Secondly, some participants expressed their concerns about the drinking habits of family and friends. The training was viewed as having increased the participants' awareness of the excessive alcohol consumption levels or possible signs of ARBD amongst individuals that participants knew personally outside their workplace. Many participants also expressed that their awareness and understanding of ARBD had been impacted by their own personal interest in the topic, and how relevant the training program was for them personally. These reports often coincided with comments relating to societal views towards alcohol and the ease of access of alcohol. Participants often expressed that alcohol was viewed as being socially acceptable and that drinking formed a natural part of their lives, with alcohol being used to celebrate, to commiserate or being associated with sporting events.

‘*I know in this country we accept that people drink … it's socially acceptable, it's not an illegal thing to do. Alright, there are supposed to be age constraints to drinking, but you know within some families it is totally acceptable for younger people to be drinking*’.

### 3.2 How successful is the ARBD training program in increasing the participants self‐reported ability to identify the signs and symptoms of ARBD, and in improving their understanding of treatment options for ARBD?

Results from the pre‐ and post‐self‐reported measures found that trainees reported a significant increase in their ability to identify signs and symptoms of ARBD following the training *t*(809) = 47.11, *p* < 0.001. After the training, an increase in the understanding of treatment possibilities was also found for the self‐reported ratings *t*(809) = 52.07, *p* < 0.001. These findings were supported by participant reports during the evaluation interviews. For example, a number of participants reported that they had identified service users within their service who may have ARBD after completing the training. Many commented that they had been able to identify signs and symptoms that were consistent with ARBD.

‘*We did actually talk about this training, and I think out of all the training we've done, this was the one we actually said “well, do you think this gentleman we support has this” and I said “yeah” so we have actually used it so it was really good*’.

As a result, some stated that they had since been able to signpost service users to further information and support. More specifically, participants reported referring service users to specialist services, encouraging the service user to seek support from their general practitioner, and discussing the issue with other team members who hold managerial roles. Relatedly, it was noted that there are limitations to the extent of involvement possible for some staff in their role. These limitations included not being trained medical professionals, meaning that their capability to correctly identify and care for someone with ARBD was limited and that there was consequently a need for service users to receive specialist medical support.

*‘I'd have to ask them if they've got any concerns or any you know, health issues that they're worried about the need they feel they need to discuss and address with the doctor, but I couldn't say to them right OK I think you've got this’*.

Many reported that the training was very relevant to their day‐to‐day work due to the nature of the service that they work in. As the training was rolled out during the COVID‐19 pandemic, the participants often discussed how this made the training particularly timely within their role as they had observed an increase in the number of service users struggling with their alcohol intake. Not only had the COVID‐19 pandemic affected service users but also participants in their role supporting clients, with adaptations being required to continue to support clients in the safest way possible. Participants also commented on how the pandemic might have impacted drinking habits which might mean that identifying signs and symptoms of ARBD is even more relevant to their role in the future.

‘*I think this COVID is … I think there'll be a spike* [in ARBD] *after this year for sure, because it's not just the isolation but its revealed to people, probably how much they could save, but how much more they could spend on alcohol still saving amount of money instead of going to the pub*’.

Participants noted that they had developed a new awareness of ARBD that related to their own professional awareness within but also outside of their immediate support role.

### 3.3 How effective is the ARBD training program in enhancing participants' confidence in supporting someone with ARBD?

Analyses of participants' confidence in supporting service users with possible ARBD demonstrated a significant increase in self‐reported ratings *t*(809) = 39.94, *p* < 0.001. Participants commented during the evaluation interviews that undertaking the training program had made them re‐evaluate some of their service users and that they had a more compassionate view towards them as a result of having a better understanding of their condition and being more tolerant of their issues.

*‘Yeah, it's like even now I can see a couple of people with it and it's like very … it is the forgetfulness, and it's like how can you not be remembering these things? So, it kinda made sense after I did the training … it's like I'd probably be a bit more tolerant now as to like “oh OK, so you forgot” like but yeah, I get it now, I can understand wh* y’.

Several participants also alluded to the stigma which is often associated with ARBD and alcohol use disorders. Participants reported that there are often challenges in making referrals and receiving a diagnosis for ARBD, although stigma was specifically perceived as a barrier to service users getting appropriate treatment. Some raised concerns that service users may not receive the support that they need due to stigma from medical professionals.

‘*Do you think the doctors would take it serious though like if an alcoholic goes into the surgery, would they actually take him serious enough to actually proceed with looking into it and diagnosing them 'cause otherwise we're just going to put down to “oh it's just self‐pity, yeah they're an alcoholic” … it's difficult 'cause they're going to come up against barriers all the time aren't they*’.

Although for some participants, these comments may indicate that their confidence in approaching medical professionals may be limited, the majority expressed that it had provided them with enhanced confidence in being able to support service users.

### 3.4 What perceptions do participants have of the training program?

All participants who completed the training program were asked nine further questions to ascertain the effectiveness of the program (see Supporting Information). These questions aimed to identify the extent to which the training impacted them and their work. Ninety‐six percent reported that it was very or extremely likely that they would recommend the training and 95% said that it was very or extremely likely that they would talk about the training with a colleague. In addition, 89% said that their attitude towards ARBD had changed after completing the training.

Participants who engaged in the evaluation interviews generally expressed their satisfaction with the training itself and which components were most useful to them within their role. Several participants mentioned that they were satisfied with the informative nature of the program.

‘*I've said this to* [my manager] *… but I really, really enjoyed the course, I commented to her I really enjoyed, I found it really good really informative it was, it was great*’.

Participants also commented on the general tone and level of complexity of the training. These comments included discussions on the accessibility of the training program and the clarity of the language used, particularly given that ARBD is a fairly complex condition with several conditions falling under the ARBD umbrella. Participants reflected on the successful online mode of delivery for the training and how this would compare had the training been delivered in person. Some also mentioned the potential utility of refresher training.

*‘I think sometimes it is very hard to do something when you're individually learning, like training like that because you don't have an opportunity to discuss things or you know, go over things with the trainer or anything, but I actually found it very helpful resource of information … so yeah, it was a positive experience for me’*.

Some participants expressed how useful it was to them to have up to date information such as statistics which gave them a better understanding of ARBD.

*‘Statistics that are relevant to our area in South Wales, Wales or whatever, just to turn around and reinforce whatever argument you're going to put across to somebody, just to run around and say well these are the statistics’*.

As the training was produced by the University and informed by research findings, some participants commented that this assisted them in supporting service users as they had been more prepared to listen to information provided by the University as opposed to an individual in a care or support role.

*‘It's all well and good me saying to them, you know these are the dangers, but, maybe if it came from somewhere educational, like the University, they might see the seriousness of it’*.

Finally, some participants commented that there was a need to expand the availability of the training further so that more people could potentially benefit from increased awareness of ARBD.

## 4 DISCUSSION

The results of this mixed‐method study found that the online training program for health‐ and social‐care workers in a housing organisation resulted in meaningful changes to participants' ARBD knowledge, attitudes, awareness and confidence. In this study, an evidence‐based training program was developed and evaluated by the present research team with the findings illustrating that evidence‐based training programs for ARBD are achievable and can have substantial and direct benefits to frontline staff. This is notable as previous research reports that current understanding of ARBD amongst frontline staff may be low [10] and that increasing awareness of ARBD is a fundamental step in improving outcomes for people living with the condition [9].

The qualitative aspect of the study supported the self‐reported quantitative ratings of awareness and understanding and provided additional information about the outcomes of the training which demonstrated that these went beyond awareness of ARBD within the workplace. Participants reported an increase in awareness of participants' own alcohol use and that of friends and family. This in‐depth exploration also highlighted that the concept of ARBD was novel for many staff members, and that several participants had already begun to relate the training to individuals that they support. This demonstrates that receiving the training resulted in wider benefits to the participants that extended beyond their immediate support role. Other key findings included changes to participants' attitudes and confidence with both the self‐reported ratings and the interviews finding that participants felt more compassionate and understanding towards the needs of individuals who may have ARBD. Stigma and negative attitudes towards alcohol use disorders in general have been highlighted in previous literature as a significant barrier to treatment [28], and therefore, the outcomes of the training program provides a promising means to begin addressing these.

Several factors relating to effective training delivery were noted by participants which included the research‐informed nature of the training, the clarity of the training, as well as its online delivery. At the same time, participants expressed that it would be beneficial if the training was available across all sectors including general practitioners, as it was noted that support services are limited in what they can do when it comes to assessing, diagnosing and supporting those with ARBD. It was also noted that refresher training would be welcomed, as would the opportunity to receive in‐person skills‐based training, given that the present training and evaluation was conducted fully online as it was undertaken during the COVID‐19 pandemic. This might subsequently facilitate and promote longer‐lasting changes to professional practice.

To date, no other training program for ARBD has been reported or evaluated, meaning that it is not possible to draw any comparisons between this program and other programs. Similar programs have been developed and evaluated for other conditions. For example, Lamph et al., [29] evaluated a training program designed to increase awareness of personality disorders, which documented significant changes after participants engaged in a multi‐agency designed training program. Several measures were reported such as increases in participants' knowledge, attitudes and capabilities, although no qualitative exploration was undertaken in their study. As a result, the current study provides supporting evidence that collaborative training programs can be successful, specifically in relation to ARBD.

### 4.1 Strengths and limitations

The strengths of this training evaluation included the evidence‐informed training approach and substantial sample size. As the training was developed by a research team who have expertise in the field, the e‐Learning program was informed by and included detailed information that was based on up‐to‐date understanding of ARBD. In addition, the mixed method nature of the study also meant that a detailed understanding of trainees' experiences could be captured which included both quantitative, measurable changes to self‐reported awareness and understanding, combined with an in‐depth understanding of experiences implementing this knowledge in support settings.

Although this study has several strengths, several limitations should also be considered. First, it is unclear whether the changes reported here will be maintained in the longer term. In the current study, self‐report measures provided evidence that there was an increase in several measures immediately following the training. Follow‐up interviews were conducted after around 10 weeks, suggesting that these changes were maintained in the short‐term, although longitudinal studies are needed to evaluate longer‐term maintenance of training benefits. Additionally, as self‐reported measures were used, these only captured perceptions of changes to awareness and knowledge. Further exploration is needed to identify if there had been changes on the measures of interest in practice.

Relatedly, participants reported enhanced information seeking behaviour and signposting to other services, but it is not yet clear if this will result in an actual increase in the appropriate diagnosis and treatment of ARBD. The recently published Welsh Government Substance Misuse Treatment Framework for ARBD [20] recommends the development of treatment pathways, and once established these will rely on an ARBD‐informed wider workforce. Impacts relating to the service users themselves was outside of the scope of this research, meaning that further exploration of any associated implications is required. One possible longer‐term implication includes the increased cost‐effectiveness of ARBD treatment, however again, the evaluation of the cost‐effectiveness of the training program was outside of the scope of the present study. Despite these limitations, the current findings are highly encouraging and indicate that the training program led to substantial improvements in understanding for staff which may also have had wider implications at an organisational level.

## 5 CONCLUSION

This is the first training program and evaluation of its kind for ARBD. The training program was effective in impacting trainees' awareness, confidence and perceptions of ARBD. As a result of this training, it is anticipated that there will be an increase in the appropriate identification of those with, or at risk of ARBD which may lead to improved opportunities to provide early interventions and treatment. Although out of the scope of the present study, is it plausible that further development and roll‐out of similar training programs will provide a cost‐effective means of addressing ARBD, given that the cost of long‐term treatment for ARBD can be high if long‐term care is required [30]. A longer‐term evaluation of the training program and any future training for ARBD is needed to establish if the changes documented in this study remain stable, or whether these effects diminish and return to baseline levels over time.

The current training program was developed and implemented within a large housing association in Wales, however, this program should be considered as part of a wider strategy to increase awareness of ARBD as recommended in the Welsh Government's Substance Misuse Treatment Framework [20]. The outcomes of this research have wider implications within the United Kingdom and beyond, as similar treatment frameworks for ARBD are currently being developed and adopted elsewhere, following the lead of the Welsh Government. The findings reported provide a strong foundation to inform future development and delivery of ARBD training programs elsewhere in the United Kingdom and further afield.

## FUNDING INFORMATION

This work was supported by the Strategic Research Investment Fund and the project is being further supported by a grant from the Knowledge Exchange and Innovation Fund, both obtained from the Higher Education Funding Council for Wales.

## CONFLICT OF INTEREST

The second author (Gareth Roderique‐Davies) was previously a non‐executive board member of the partner organisation until 2017 but received no remuneration for this role. The research underpinning the training was part‐funded by the partner organisation, however, this underpinning research was completed prior to the development of the training program. No additional financial investment or benefit was provided from the organisation for the purpose of developing this training program or undertaking the evaluation.

## Supporting information


**Data S1** Supporting information

**Data S2** Supporting information

**Data S3** Supporting information
