# Supplementary material for: Alcohol‐related brain damage: A mixed‐method evaluation of an online awareness‐raising programme for frontline care and support practitioners
Source: Drug Alcohol Rev. 2022 Sep 12;42(1):46–58. doi: 10.1111/dar.13545 (PMC10087889; doi:10.1111/dar.13545)
Supplement: Supplementary file 1 — Data S1 Supporting information [file DAR-42-46-s001.pdf]

Pre-Training Questionnaire

What is your role?

- Frontline worker
- Supervisor
- Team leader
- Manager
- Area Manager
- Other

If you selected 'other' please specify:

Are you:

- Male
- Female
- Other
- Prefer not to say

Which sector/service do you work in?

____

Before this training, had you heard of ARBD?

- Yes
- No

Please rate the following:

	Poor	*Below Average*	*Average*	*Above Average*	*Good*
Your awareness of ARBD					
Your understanding of ARBD					
Your ability to identify someone at risk of ARBD					
Your ability to identify the signs and symptoms of ARBD					
Your understanding of treatment possibilities for ARBD					
Your confidence in supporting someone with ARBD					

Post-Training Questionnaire

What is your role?

- Frontline worker
- Supervisor
- Team leader
- Manager
- Area Manager
- Other

If you selected 'other' please specify:

Are you:

- Male
- Female
- Other
- Prefer not to say

Which sector/service do you work in?

____

Has your attitude towards ARBD changed?

- Yes
- No

If your awareness, understanding or attitude towards ARBD have changed as a result of this training, please explain:

____

Please rate the following:

	Poor	*Below Average*	*Average*	*Above Average*	*Good*
Your awareness of ARBD					
Your understanding of ARBD					
Your ability to identify someone at risk of ARBD					
Your ability to identify the signs and symptoms of ARBD					
Your understanding of treatment possibilities for ARBD					

Your confidence in supporting someone with ARBD	

How likely is it that you will:

	Not at all likely	*Not so likely*	*Unsure*	*Very likely*	*Extremely likely*
Recommend this training to a colleague?					
Talk about this training with a colleague?					
Talk about this training with a friend/family member?					

How relevant was the training for your role?

- Extremely relevant
- Very relevant
- Somewhat relevant
- Not so relevant
- Not relevant at all

In your opinion, what was the best part of the training?

____

Can you name one thing that you will take away from the training that you can implement in your role?

____

Do you have any further training needs for ARBD?

- Yes
- No

Do you have any suggestions for future research? If so, please list these below:

____

Do you have any other comments or questions? If so, please leave these below:

____
