# Supplementary material for: Alcohol‐related brain damage: A mixed‐method evaluation of an online awareness‐raising programme for frontline care and support practitioners
Source: Drug Alcohol Rev. 2022 Sep 12;42(1):46–58. doi: 10.1111/dar.13545 (PMC10087889; doi:10.1111/dar.13545)
Supplement: Supplementary file 2 — Data S2 Supporting information [file DAR-42-46-s002.pdf]

University of South Wales
Prifysgol De Cymru

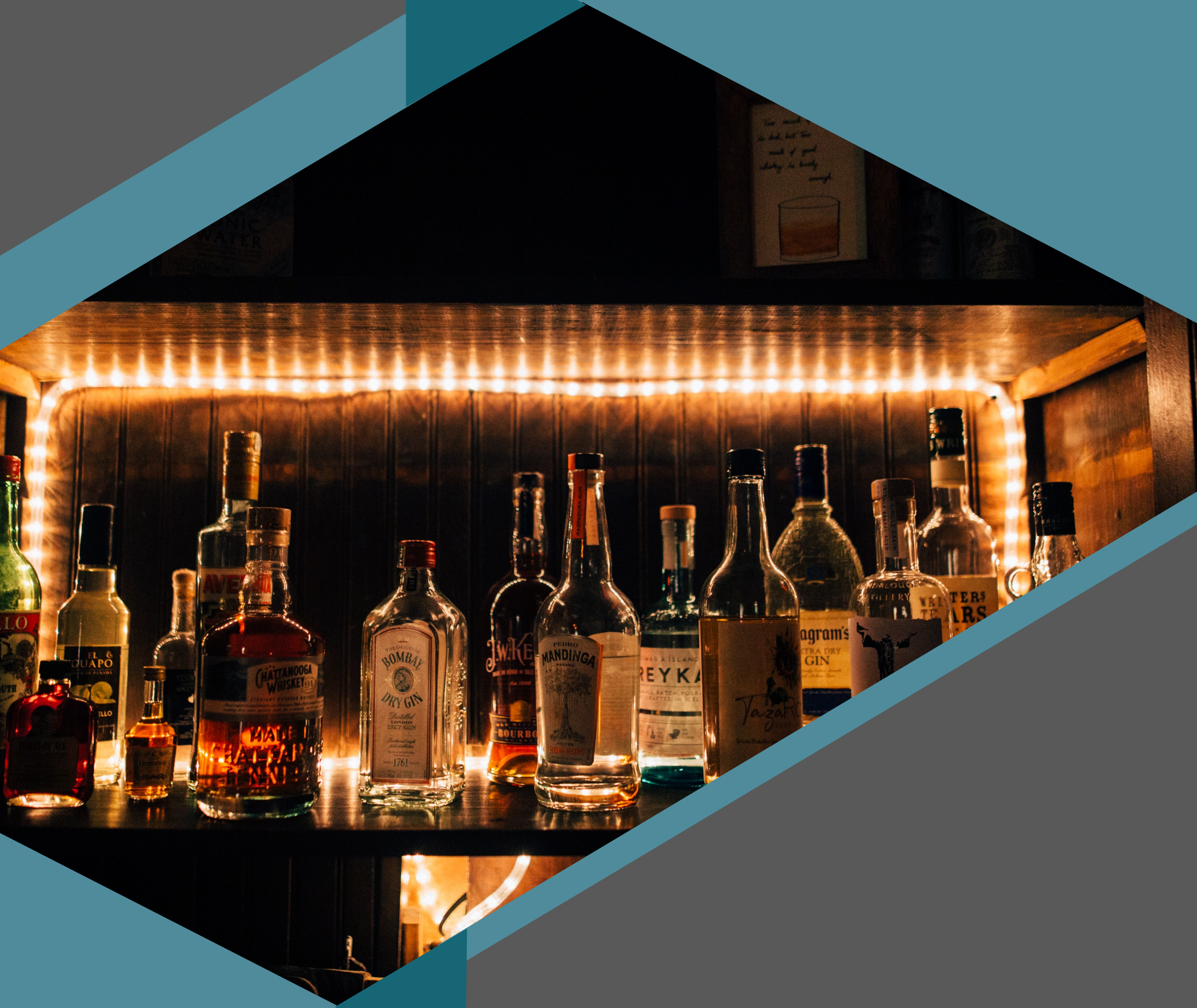

ARBD

Training Summary

University of South Wales
Prifysgol De Cymru

Increasing Awareness and Understanding of Alcohol-Related Brain Damage

Alcohol-Related Brain Damage (ARBD) is an under-recognised condition that is caused by chronic alcohol misuse and associated nutritional deficiencies. It is a significant public health concern both in community and clinical settings, yet there is a lack of knowledge and understanding of ARBD among health and social care professionals across a range of settings.

> "Alcohol-Related Brain Damage is a significant public health concern both in community and clinical settings"

The need to increase awareness of ARBD has been highlighted in the Welsh Government's draft treatment framework for ARBD, in which the first tier of the recommendations is for basic training of ARBD for all professionals who work with individuals with, or who are at risk of ARBD. The lack of awareness of this condition has also been identified by the Royal College of Psychiatrists as a barrier to improving the care and support of those with the condition.

As a result, the aim of this project was to implement the first recommendation of this treatment framework for ARBD by improving awareness of the condition amongst a variety of stakeholders. This project was conducted in collaboration with a large housing organisation who are the largest not for profit housing association in Wales who have significant arms in care and support services throughout Wales.

A bespoke training package was developed to meet these aims by increasing care and support staff's confidence, awareness and knowledge of ARBD. This multi-level training package was underpinned our extensive research findings and expertise that has been amassed through consultations with clinicians, third sector providers and service users across Wales, as well as with our expert external collaborators, Public Health Wales and Alcohol Change UK. This summary document serves to highlight the range of training resources available and the outcomes of the training to date.

For further information, visit the **Addictions Research Group** Web Pages here. To visit the **ARBD Wales Website,** see here.

University of South Wales Prifysgol De Cymru

E-Learning Training Modules

The training is made up of two e-learning modules. Each module takes around 25 minutes to complete and has three sections. The content focusses on identifying ARBD, the prevalence and characteristics of ARBD, and the importance of treatment and early intervention.

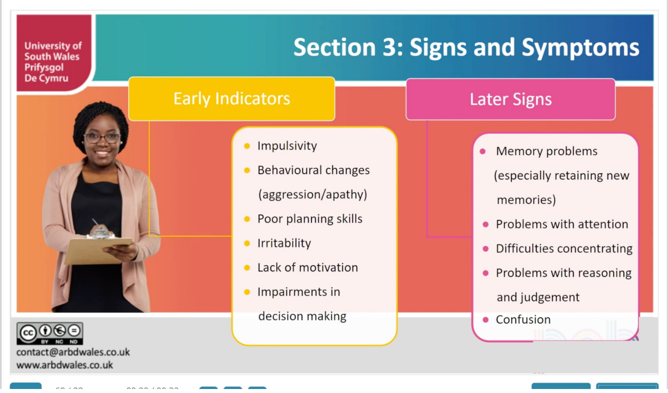

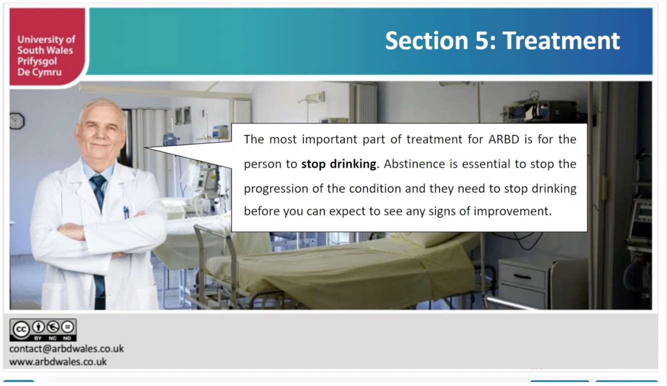

The e-learning modules are self guided and have an optional voiceover. There is an in-built evaluation process which captures important information about learners prior and post awareness, understanding and attitudes towards ARBD.

These modules contain a variety of features, including a range of interactive elements, scene-based slides and information sections. Alongside the e-learning, there are a range of additional resources including infographics, handbooks and information leaflets.

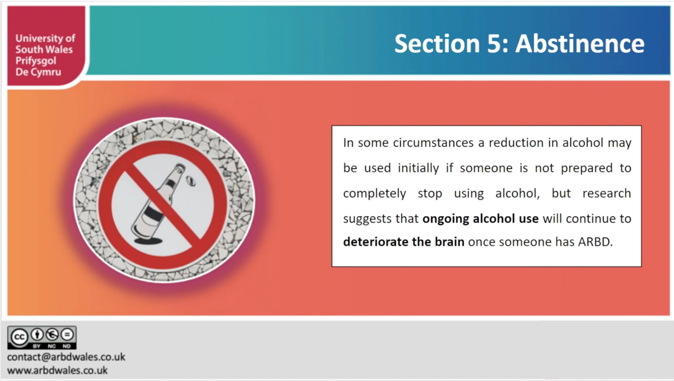

Resources

University of South Wales
Prifysgol De Cymru

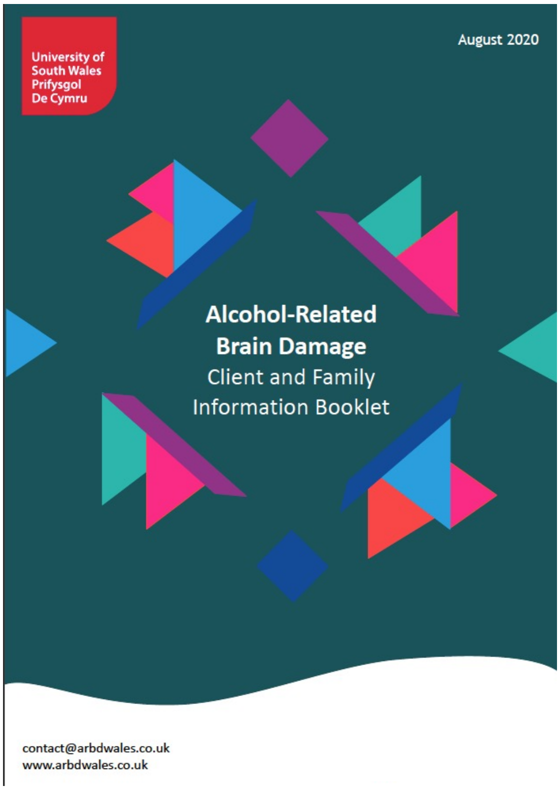

University of South Wales
Prifysgol De Cymru

August 2020

Alcohol-Related Brain Damage
Client and Family Information Booklet

contact@arbdwales.co.uk
www.arbdwales.co.uk

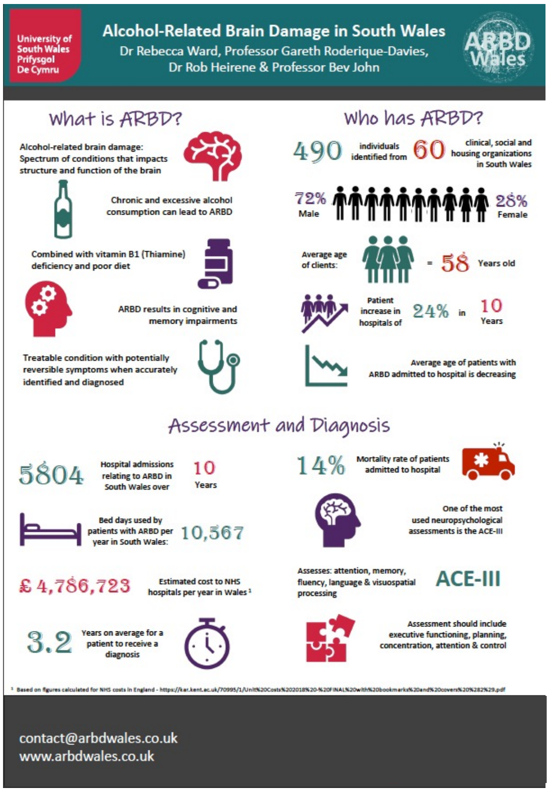

University of South Wales
Prifysgol De Cymru

Alcohol-Related Brain Damage in South Wales

Dr Rebecca Ward, Professor Gareth Roderique-Davies, Dr Rob Heirene & Professor Bev John

ARBD Wales

What is ARBD?

Alcohol-related brain damage: Spectrum of conditions that impacts structure and function of the brain

Chronic and excessive alcohol consumption can lead to ARBD

Combined with vitamin B1 (Thiamine) deficiency and poor diet

ARBD results in cognitive and memory impairments

Treatable condition with potentially reversible symptoms when accurately identified and diagnosed

Who has ARBD?

490 individuals identified from 60 clinical, social and housing organizations in South Wales

72% Male 28% Female

Average age of clients: = 58 Years old

Patient increase in hospitals of 24% in 10 Years

Average age of patients with ARBD admitted to hospital is decreasing

Assessment and Diagnosis

5804 Hospital admissions relating to ARBD in South Wales over 10 Years

Bed days used by patients with ARBD per year in South Wales: 10,567

£ 4,786,723 Estimated cost to NHS hospitals per year in Wales [1]

3.2 Years on average for a patient to receive a diagnosis

14% Mortality rate of patients admitted to hospital

One of the most used neuropsychological assessments is the ACE-III

Assesses: attention, memory, fluency, language & visuospatial processing ACE-III

Assessment should include executive functioning, planning, concentration, attention & control

[1] Based on figures calculated for NHS costs in England - https://kar.kent.ac.uk/70995/1/Unit%20Costs%202018%20-%20FINAL%20with%20bookmarks%20and%20covers%20%282%29.pdf

contact@arbdwales.co.uk
www.arbdwales.co.uk

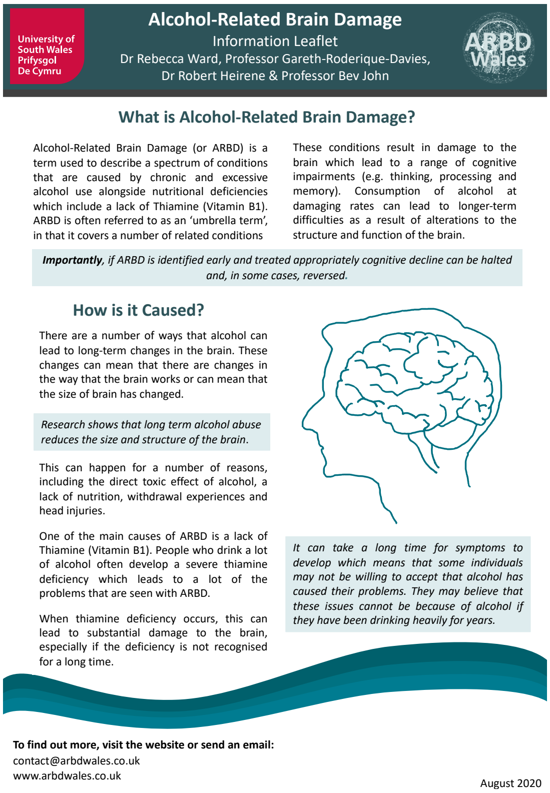

University of South Wales
Prifysgol De Cymru

Alcohol-Related Brain Damage

Information Leaflet
Dr Rebecca Ward, Professor Gareth Roderique-Davies, Dr Robert Heirene & Professor Bev John

ARBD Wales

What is Alcohol-Related Brain Damage?

Alcohol-Related Brain Damage (or ARBD) is a term used to describe a spectrum of conditions that are caused by chronic and excessive alcohol use alongside nutritional deficiencies which include a lack of Thiamine (Vitamin B1). ARBD is often referred to as an 'umbrella term', in that it covers a number of related conditions

These conditions result in damage to the brain which lead to a range of cognitive impairments (e.g. thinking, processing and memory). Consumption of alcohol at damaging rates can lead to longer-term difficulties as a result of alterations to the structure and function of the brain.

***Importantly**, if ARBD is identified early and treated appropriately cognitive decline can be halted and, in some cases, reversed.*

How is it Caused?

There are a number of ways that alcohol can lead to long-term changes in the brain. These changes can mean that there are changes in the way that the brain works or can mean that the size of brain has changed.

Research shows that long term alcohol abuse reduces the size and structure of the brain.

This can happen for a number of reasons, including the direct toxic effect of alcohol, a lack of nutrition, withdrawal experiences and head injuries.

One of the main causes of ARBD is a lack of Thiamine (Vitamin B1). People who drink a lot of alcohol often develop a severe thiamine deficiency which leads to a lot of the problems that are seen with ARBD.

When thiamine deficiency occurs, this can lead to substantial damage to the brain, especially if the deficiency is not recognised for a long time.

It can take a long time for symptoms to develop which means that some individuals may not be willing to accept that alcohol has caused their problems. They may believe that these issues cannot be because of alcohol if they have been drinking heavily for years.

To find out more, visit the website or send an email:
contact@arbdwales.co.uk
www.arbdwales.co.uk

August 2020

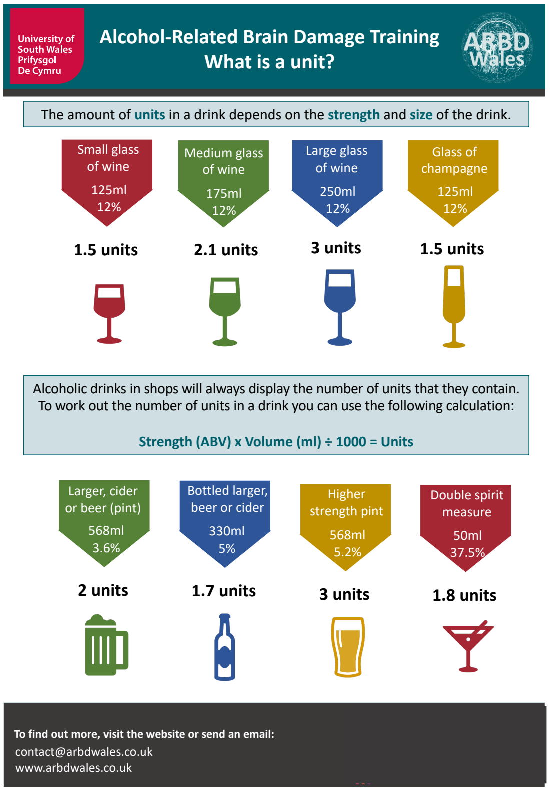

University of South Wales
Prifysgol De Cymru

Alcohol-Related Brain Damage Training
What is a unit?

ARBD Wales

The amount of **units** in a drink depends on the **strength** and **size** of the drink.

Small glass of wine	Medium glass of wine	Large glass of wine	Glass of champagne
125ml 12%	175ml 12%	250ml 12%	125ml 12%
1.5 units	2.1 units	3 units	1.5 units

Alcoholic drinks in shops will always display the number of units that they contain. To work out the number of units in a drink you can use the following calculation:

Strength (ABV) x Volume (ml) ÷ 1000 = Units

Larger, cider or beer (pint)	Bottled larger, beer or cider	Higher strength pint	Double spirit measure
568ml 3.6%	330ml 5%	568ml 5.2%	50ml 37.5%
2 units	1.7 units	3 units	1.8 units

To find out more, visit the website or send an email:
contact@arbdwales.co.uk
www.arbdwales.co.uk

Supplementary Videos

Alongside these are three supplementary videos from specialists who have experience in working in the ARBD field. These include:

Dr Julia Lewis: Consultant Addiction Psychiatrist and Clinical Lead in the Aneurin Bevan University Health Board

Dr Raman Sakhuja: Consultant Psychiatrist specialising in Addiction Psychiatry and General Adult Psychiatry, Cwm Taf University Health Board

Andrew Misell: Director for Wales, Alcohol Change UK

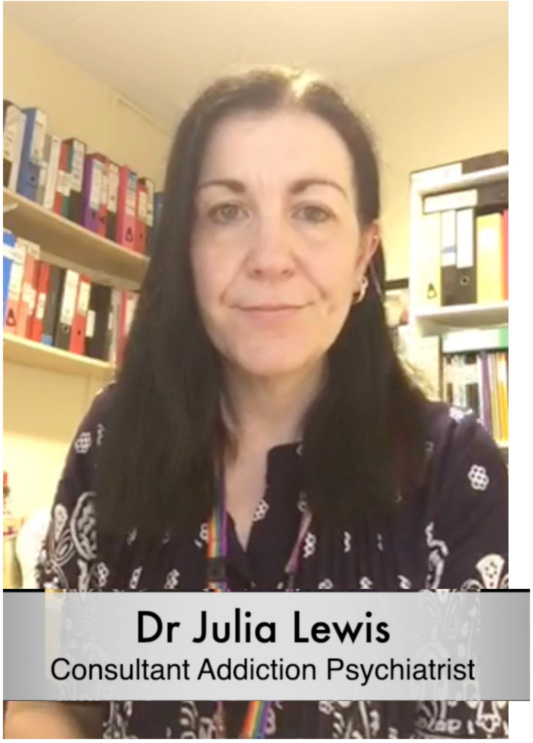

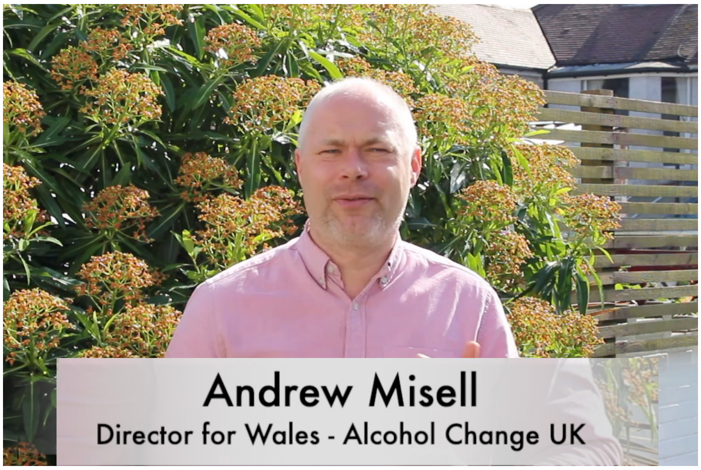

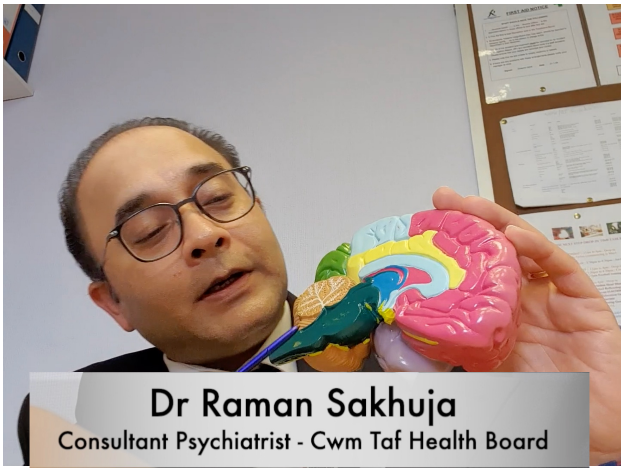

University of South Wales
Prifysgol De Cymru

Wales Alcohol-Related Brain Damage Website

Finally, an information website has been created which is compatible on computers, tablets and mobiles. The website address is http://www.arbdwales.co.uk/ and it includes a number of pages with information about ARBD as well as links to some of the resources.

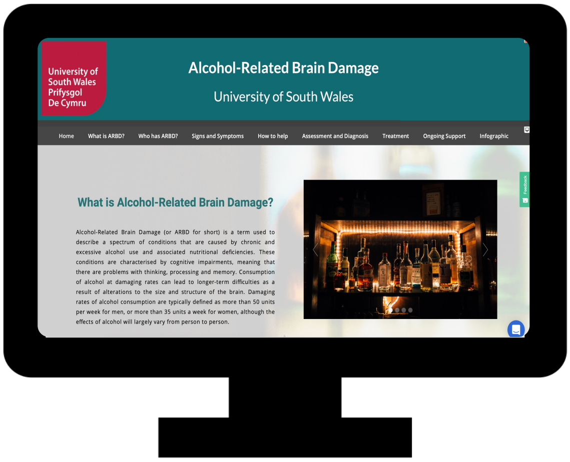

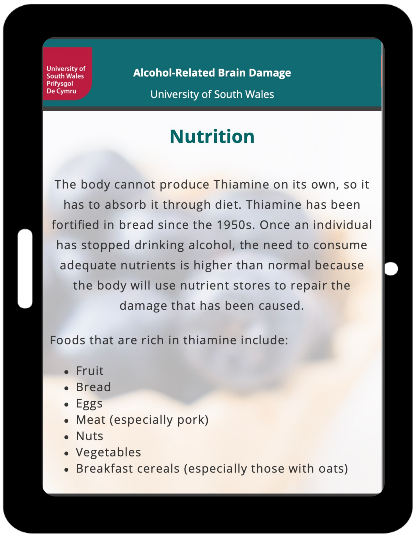

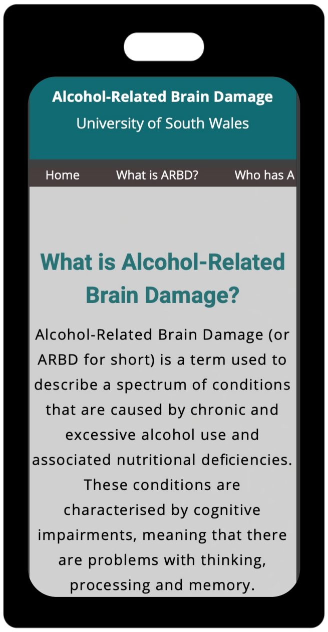

Outcomes

To date, the training has been launched across support services within the housing organisation. Four hundred and seventy staff members have enrolled on the training since it launched four weeks ago. Of these, 87% have completed all elements of the training which includes the e-learning, videos and accessing the further resources presented earlier.

The outcomes from the training as reported by trainees is presented in this section. These include information relating to how useful trainees have found the training, its applicability across services and direct quotes from those who have completed the training.

It would be a joyous moment to see this research and strategies rolled out equally in Wales. Apart from that, I gained a huge leap of knowledge for my clients.

Training Satisfaction

97%

Said it was **"very likely"** or **"extremely likely"** that they would recommend the training.

Change in Awareness and Understanding of ARBD

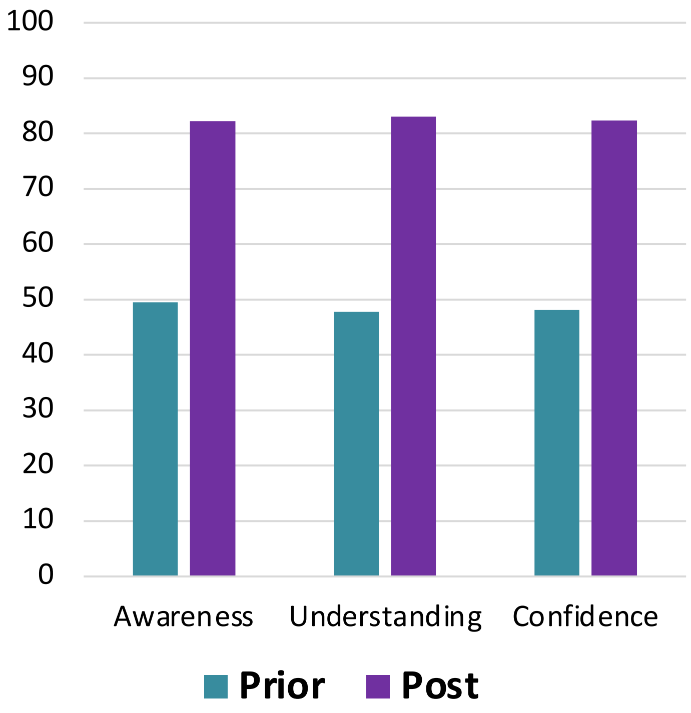

I realise that I have at least one client who may have or be at risk of developing ARBD - my approach to support will take this on board and I understand the need to repeat activities or goals with my client more times than I would with someone who does not suffer from this condition.

Outcomes

University of South Wales
Prifysgol De Cymru

The training has improved my knowledge and understanding of ARBD and has given me a better insight in how to support clients with ARBD and what can be put in place to help the individual.

Training Impact

96%

Said it was **"very likely"** or **"extremely likely"** that they would talk about the training with a colleague.

Prior Awareness of ARBD

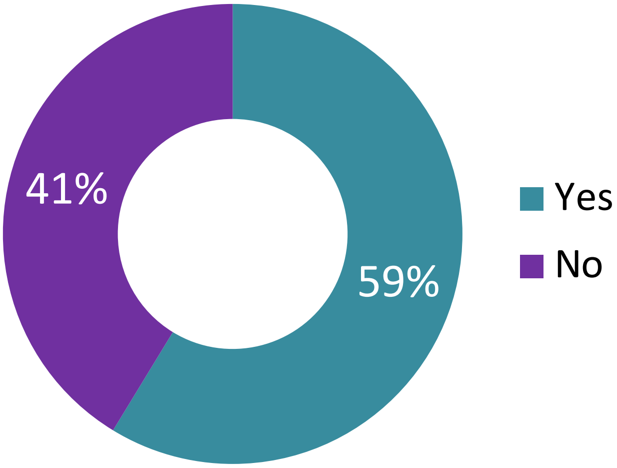

I am definitely going to be more aware of this condition and have a much better idea of what I can do to help or support someone with ARBD.

499 **EMPLOYEES**

enrolled on the training course in the first 2 and a half months of launch

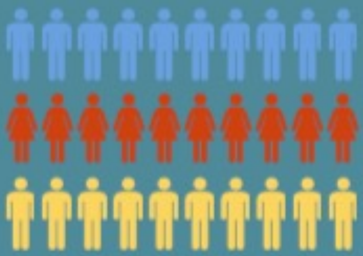

University of South Wales
Prifysgol De Cymru

Attitude towards ARBD

89%

Said it that their **attitude towards ARBD had changed** after completing the training.

> I now understand a lot more about ARBD and have identified some of my clients that may be suffering from it.

> I knew very little about ARBD so my understanding, attitude and awareness has greatly increased. I found the course very interesting and informative. I will be more able to look for the signs of ARBD when supporting my clients.

Trainee Role

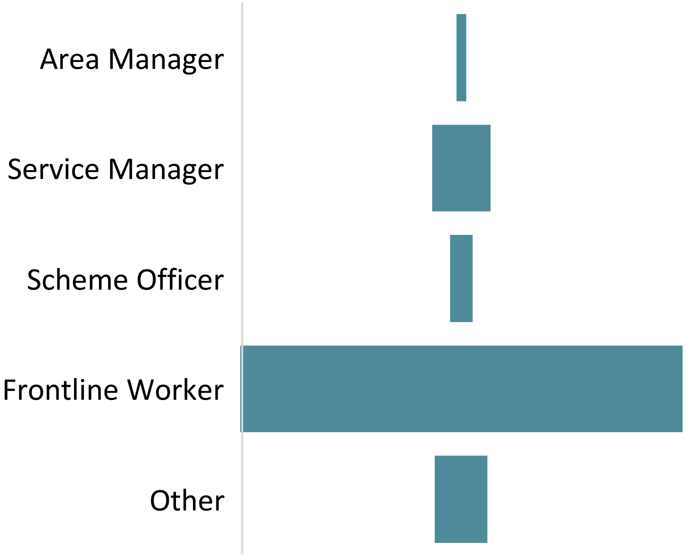

Confidence

83%

of staff reported that their confidence in supporting individuals with ARBD had increased.

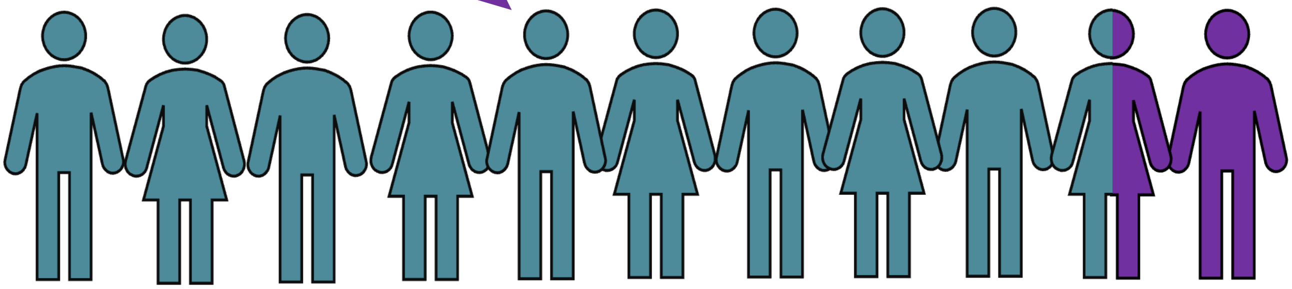

88% of staff to date have completed the full training course

Acknowledgements

The researchers at the University of South Wales would like to extend their gratitude to the professionals and organizations that have assisted and supported the development and implementation of these training materials. We are especially grateful to the following individuals for their input in developing these resources:

Dr Robert Heirene: Postdoctoral Research Associate at the Gambling Treatment & Research Clinic, The University of Sydney.

Dr Julia Lewis: Consultant Addiction Psychiatrist and Clinical Lead, Aneurin Bevan University Health Board.

Dr Raman Sakhuja: Consultant Psychiatrist specialising in Addiction Psychiatry and General Adult Psychiatry, Cwm Taf University Health Board.

Andrew Misell: Director for Wales, Alcohol Change UK.

Visit the **Addictions Research Group** Web Pages here.

Visit the **ARBD Wales Website** here.

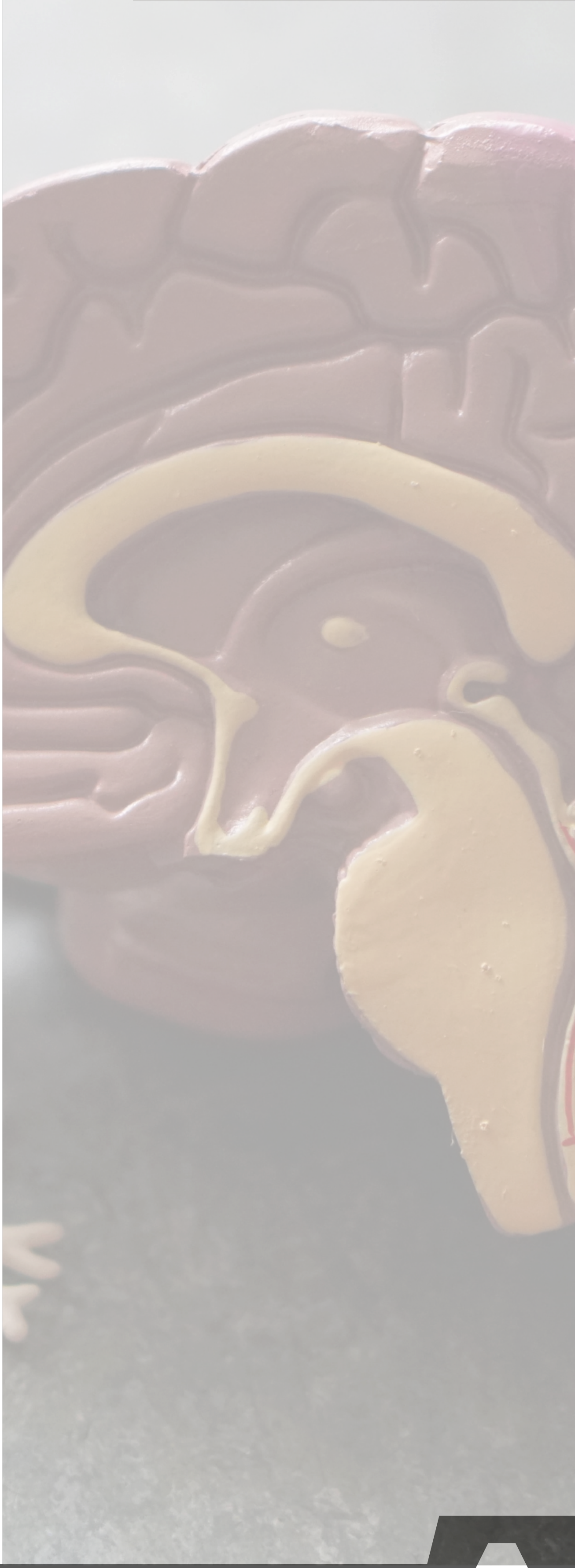

Alcohol-Related Brain Damage

Increasing Awareness and Understanding

The project was undertaken by:

Dr Rebecca Ward is a Senior Research Assistant in Psychology at the University of South Wales. Her main focus is on developing a training package in partnership with third sector organisations to increase awareness of Alcohol-Related Brain Damage. The focus of this project is to utilise research findings in a novel way to inform the practice within the health and social care sector.

Professor Gareth Roderique-Davies is Professor of Psychology at the University of South Wales with expertise in substance misuse, behavioural addiction, craving and the long-term effects of recreational drug use. He's an HCPC-registered Psychologist, a Chartered Psychologist and an Associate Fellow of the British Psychological Society.

Professor Bev John is Professor of Addictions and Health Psychology at the University of South Wales. She has worked in the field of psychological health for many years, in research, teaching and treatment delivery, developing and evaluating health related interventions. She has also delivered psychological therapies. Her main focus is applied research in psychological health, in particular promoting positive behaviour change in mental and physical health and substance misuse; and the development and evaluation of psychological interventions.

Visit the **Addictions Research Group** Web Pages here.

Visit the **ARBD Wales Website** here.
