# Supplementary material for: Alcohol‐related brain damage: A mixed‐method evaluation of an online awareness‐raising programme for frontline care and support practitioners
Source: Drug Alcohol Rev. 2022 Sep 12;42(1):46–58. doi: 10.1111/dar.13545 (PMC10087889; doi:10.1111/dar.13545)
Supplement: Supplementary file 3 — Data S3 Supporting information [file DAR-42-46-s003.pdf]

Interview/Focus Group Schedule
Evaluating the Impact of an Online Awareness Raising Training Programme for ARBD

Introduction:

- Thank participants for agreeing to take part
- Remind participants that everything they say will be anonymous but that they should not refer to any clients by name
- Remind participants that the focus groups/interviews will be recorded for transcribing and that they have the right to withdraw at any point
- Ask each person to introduce themselves by stating their role and the type of service that they work in

Training Specific:

- Did you all manage to access the training course for ARBD?
- How did you find the training?
    - How accessible was the training?
    - What did you think of the contents, presentation and format of the training?
    - Was there anything missing from the training that you would have found beneficial?
- Would you change anything about the training?

Prior Awareness:

- Had you heard of ARBD before?
    - Umbrella term – had they heard of any other terminology?
- Have you ever known or supported someone with ARBD?
    - In what context and how did they find this?
- How did you rate your awareness of ARBD before the training?

Post Awareness:

- Has your awareness of ARBD changed?
    - If so, how?
- Has this impacted the way that you work in any way?
- Has your attitude towards ARBD changed?
    - How has your attitude changed?
    - Would you approach a client with ARBD in a different way?
- How confident do you feel in recognising the signs of ARBD?
    - Has this changed since completing the training?
    - What signs might you notice?
    - Would you feel comfortable in speaking to someone about making a referral?
- What further support might you need?

General:

- Would you like to know more about ARBD in the future?
    - If so, what would you like to know?
- Are there any other issues that you think need addressing in this field?
    - If so, what are these issues and how might they be addressed?
- Is there anything else you'd like to add or that we haven't covered?

Close:

- Thank participants for taking part
- Remind participants that they can get in touch if they have any issues or questions
